# Numerical Simulation and Machine Learning Prediction of the Direct Chill Casting Process of Large-Scale Aluminum Ingots

**DOI:** 10.3390/ma17061409

**Published:** 2024-03-19

**Authors:** Guanhua Guo, Ting Yao, Wensheng Liu, Sai Tang, Daihong Xiao, Lanping Huang, Lei Wu, Zhaohui Feng, Xiaobing Gao

**Affiliations:** 1 National Key Laboratory of Science and Technology on High-Strength Structural Materials, Central South University, Changsha 410083, China; 213312083@csu.edu.cn (G.G.); liuwensheng@csu.edu.cn (W.L.); christie@csu.edu.cn (L.H.); 2 Shaanxi Nonferrous Yulin New Material Group Co., Ltd., Yulin 719099, China; dinglangkuangdang@163.com (T.Y.); gaoxb0778@sohu.com (X.G.); 3 Beijing Engineering Research Center of Advanced Aluminum Alloys and Applications, Beijing Institute of Aeronautical Materials, Beijing 100095, China; wl5ra@126.com (L.W.); fzhh1991@sina.com (Z.F.)

**Keywords:** direct chill casting, solidification, finite element analysis, machine learning, process optimization, aluminum alloys

## Abstract

The large-scale ingot of the 7xxx-series aluminum alloys fabricated by direct chill (DC) casting often suffers from foundry defects such as cracks and cold shut due to the formidable challenges in the precise controlling of casting parameters. In this manuscript, by using the integrated computational method combining numerical simulations with machine learning, we systematically estimated the evolution of multi-physical fields and grain structures during the solidification processes. The numerical simulation results quantified the influences of key casting parameters including pouring temperature, casting speed, primary cooling intensity, and secondary cooling water flow rate on the shape of the mushy zone, heat transport, residual stress, and grain structure of DC casting ingots. Then, based on the data of numerical simulations, we established a novel model for the relationship between casting parameters and solidification characteristics through machine learning. By comparing it with experimental measurements, the model showed reasonable accuracy in predicting the sump profile, microstructure evolution, and solidification kinetics under the complicated influences of casting parameters. The integrated computational method and predicting model could be used to efficiently and accurately determine the DC casting parameters to decrease the casting defects.

## 1. Introduction

Large-scale aluminum ingot is an indispensable raw material for the fabrication of large components widely used in rail transportation, aerospace, shipbuilding, and other industry fields [1]. Compared with the permanent mold casting, direct chill (DC) casting is much more productive due to the assembly line of casting [2]. Nowadays, DC casting has already become the most important and productive approach for the massive production of steel and aluminum ingots [3].

**Figure 1:**
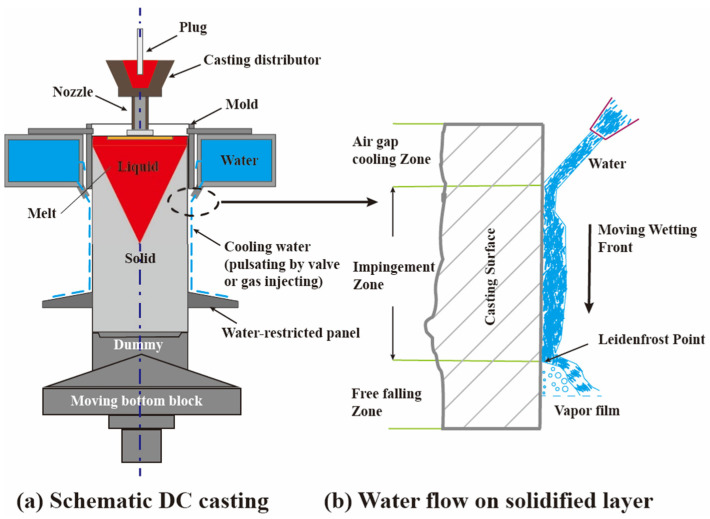
Schematic diagram of DC casting process.

The DC casting produces aluminum slabs or ingots semi-continuously by pulling a solidified ingot downward as shown in Figure 1 [4]. The solidification process of DC casting is highly intricate and controlled by the interactions between multiple physics fields, such as temperature, concentration, flow, and stress fields [5]. The evolution of these physical fields during the actual casting process depends on the strict control of numerous input parameters. Minor changes to the DC casting recipe, like pouring temperature or casting velocity, can significantly impact the casting outcome and lead to defects [6]. Consequently, scientists and foundry engineers [7] have developed several mathematical models for input–output relationships to accelerate the optimization of casting recipes instead of relying on abundant trial-and-error DC casting experiments.

Preventing casting defects is also very difficult for the DC casting process of aluminum alloys. Generally, controlling the defects of the DC casting process for aluminum alloys is more difficult than for that of steel. This is due to the solidification range of aluminum alloy being much wider than that of steel, comparing the temperature range of approximately 80 °C for steel solidified at about 1400 °C [8] with a temperature range of about 120 °C for aluminum alloy solidified at about 600 °C [4]. The depths of mushy zones and residual stress are closely related to the formation of defects such as hot cracking. Prolonged exposure to the solid–liquid transition zone increases the vulnerability of the DC casting process for aluminum alloy. Moreover, the yield strength of as-cast aluminum alloy is lower than that of steel. Consequently, the heat stress that the ingot can withstand during the DC casting process is smaller, making it more prone to cracking. Obviously, experimental searching for the optimal solution to eliminate defects by tuning the multiple casting parameters is an extremely time- and cost-consuming task.

In recent years, numerical simulation has become an important tool for studying the evolution of ingots in the DC casting process. This offers a reliable and efficient solution for the design of large-scale aluminum casting recipes [1,4,9,10,11,12,13]. Luo et al. [11] employed the finite volume method to study the effect of different casting processes on the macro-segregation distribution of the 2024 aluminum alloy ingots. The authors found that under the condition of the same ingot size, casting speed has the most significant effect on the macro-segregation of ingots, followed by the cooling intensity of the second cooling zone, while pouring temperature has the least impact. Han et al. [12] conducted a quantitative analysis on the effects of various casting process parameters, including cooling water flow rate, withdrawal speed, and pouring temperature, on the sump morphology in the casting blank crystallizer and the eventual distribution of the structure field within the casting blank. Such previous studies have demonstrated that DC casting is a complex process involving the interactions among multi-physic fields which requires a sophisticated control of the casting parameters to produce high-quality ingots.

Even though it is still a formidable challenge to optimize the casting parameters due to the conflict between limited data points and complex multi-physics interactions, the success of machine learning (ML) algorithms has been presently drawing the attention of casting engineers due to its outstanding predictive capabilities in monitoring and controlling complex processes [14,15,16,17,18]. Combined with high-throughput numerical modeling and experiments, ML could accurately and efficiently predict the evolution complex processes affected by complex multivariables. Lee et al. [19] proposed a deep learning model to predict the temperature distribution in the steelmaking DC casting process. The results showed a significant improvement in the model’s prediction performance compared to the baseline model. Hore et al. [20] employed an artificial neural network (ANN) model to analyze the impact of various process parameters, including melt composition and casting velocity, on critical measures like metallurgical length and probability of defect occurrence during DC casting. The prediction outcomes generated by ANN were successfully validated using multiple linear regression analysis.

While there have been many studies focusing on optimizing the DC casting process for large-scale aluminum alloy ingots [21,22,23,24,25], there is still a lack of research on the impact of process parameters on the DC casting process under multi-field coupling. Furthermore, there are few reports on quantitative studies combined with machine learning to develop appropriate prediction models for large-scale aluminum alloy DC casting ingots.

This study aims to address the pressing need for an accurate and efficient prediction of the input–output relationship for the DC casting process of aluminum alloys. A three-dimensional centrosymmetric model has been utilized to explore how pouring temperatures, casting velocities, primary cooling intensities (PCIs), and secondary cooling water flow rates (SCWFRs) impact the transient physical fields during DC casting of high-strength 7xxx aluminum alloy. Based on the multi-physics field data gathered from the numerical simulation, this study quantitatively evaluated the relationship between significant physical parameters, such as sump depth, mushy zone width, and the solidified shell thickness, and varied process parameters in the DC casting process by using ML. The model is validated using experimental data from previous research, and the results demonstrate a satisfactory accuracy of prediction.

## 2. DC Model and Simulation Methods

### 2.1. Governing Equations of DC Model

#### 2.1.1. Equation of Macroscopic Transport

A single region formulation based on the conservation of mass, momentum, and energy was used to calculate the melt flow, heat transfer, and solidification in the DC process. The continuum formulation avoids the difficulty of tracking phase interfaces, while still providing reasonable results at low computational cost [1]. Furthermore, this study used transient equations instead of steady-state solutions during DC casting. This is because the transient term acts as an inertial relaxation term and aids in convergence. The solution is stopped once a stable sump profile, as indicated by the coherency isosurface, is achieved.

The mass conservation equation is given by

(1) $$\frac{\vartheta \rho }{\vartheta t}+\nabla \cdot (\rho u)=0$$

At a packing fraction ${f_{s}}^{th}$, free-floating dendrites coalesce and form a rigid dendritic structure, which divides the phase transition zone into a slurry zone and a mushy zone. And the buoyancy term is evaluated in assuming the Boussinesq approximation. The momentum conservation equation in the liquid and slurry region:

(2) $$\frac{\vartheta (\rho u)}{\vartheta t}+\nabla \cdot \left(\rho uu\right)=-\nabla p+\nabla \cdot \left\{\left(\mu +\mu_{t}\right)\nabla u\right\}+\rho_{b}g$$

In the mushy zone and solid region, the momentum conservation equation is given by

(3) $$\frac{\vartheta (\rho u)}{\vartheta t}+\nabla \cdot \left(\rho uu\right)=-\nabla p+\nabla \cdot \left\{\left(\mu +\mu_{t}\right)\nabla u\right\}+\rho_{b}g-(u-u_{s})\frac{\left(1-{f_{l}}^{2}\right)}{{f_{l}}^{3}}K_{P}\;\rho_{b}g=\rho_{ref}g[\beta_{T}\left(T-T_{ref}\right)\underset{s}{+\sum }\beta_{S}(C_{l}^{s}-C_{0}^{s})]$$

The release of latent heat is a critical feature that distinguishes the solidification process of metals from other heat transfer processes. It is determined directly by the nucleation and growth of grains in the microscopic category and furthermore provides insight into temperature field calculations. So, this paper employed the enthalpy [26] to handle the release of latent heat during solidification. The energy conservation equation [1,5,10,27] is given by

(4) $$\frac{\vartheta (\rho H)}{\vartheta t}+\nabla \cdot \left(\rho Hu\right)=\nabla \cdot \left(K\nabla T\right)-L\left\{\frac{\vartheta (\rho f_{l})}{\vartheta t}+\nabla \cdot (\rho uf_{l})\right\}$$

(5) $$H\left(T\right)=\int_{0}^{T}cdT+L(1-f_{s})$$

where ρ is the density; u is the velocity; t is the time; μ and $\mu_{t}$ are the dynamic viscosity and the turbulent viscosity of the molten metal, respectively; g is the acceleration due to gravity; p is the pressure; c is the specific heat; K is the thermal conductivity; $K_{P}$ is the permeability coefficient; H is the enthalpy; T is the temperature; L represents the release of latent heat; $f_{s}$ and $f_{l}$ are the volume fraction of solid and liquid phase, respectively; $\beta_{T}$ is the thermal expansion coefficient and $\beta_{S}$ is the solute expansion coefficient for species s; $C_{0}^{s}$ is the reference concentration value for species s.

#### 2.1.2. Constitutive Equations

In the upper region of the two-phase region (from the liquidus temperature to the linear shrinkage temperature), grains can move and rearrange relatively freely, indicating the absence of thermal stress during this stage. The mushy region, between the linear shrinkage temperature and the non-equilibrium solidus temperature, is considered a dendritic network. As a dense solid network structure forms, thermal stress begins to occur. In this temperature range, the mushy zone was treated as a visco-plastic porous medium that is filled with saturated liquid. Then, a thermoelastic-plastic model was adopted in this study. The total strain of the ingot in the thermoelastic-plastic model consists of the thermal strain (${\varepsilon_{s}}^{T}$), the elastic strain (${\varepsilon_{s}}^{\varepsilon }$), and the plastic strain (${\varepsilon_{s}}^{p}$) [28],

(6) $$\varepsilon =\frac{1}{2}\left(\nabla u_{s}+{\left(\nabla u_{s}\right)}^{T}\right)={\varepsilon_{s}}^{T}+{\varepsilon_{s}}^{\varepsilon }+{\varepsilon_{s}}^{p}$$

The elastic strain is calculated as

(7) $$\sigma ={E\varepsilon_{s}}^{\varepsilon }$$

The thermal strain can be calculated by the thermal strain rate; it is given by

(8) $${\dot{\varepsilon_{s}}}^{T}=\frac{1}{3}\psi \left(f_{s}\right)\beta_{T}\frac{dT}{d}I,\;\mathrm{with}\;\psi \left(f_{s}\right)=\left\{\begin{array}{c} 0\\ {\left(\frac{f_{s}-{f_{s}}^{th}}{1-{f_{s}}^{th}}\right)}^{n} \end{array}\right.\;\mathrm{for}\;\left\{\begin{array}{c} f_{s}\leq {f_{s}}^{th}\\ f_{s}>{f_{s}}^{th} \end{array}\right.$$

At the plastic stage, the stress satisfied the yield function $f\left(\sigma_{ij}\right)=0$, using the Prandtl–Reuss flow rule to calculate the plastic strain (detailed description can be find in [29]):

(9) $${\varepsilon_{s}}^{p}=\gamma \frac{\vartheta f}{\vartheta \sigma }$$

where f is the yield stress; γ is the plastic multiplier to be determined with the aid of the consistency condition f=0.

The constitutive equation of the AA7050 alloy below the solidus temperature is based on the extended Ludwik equation.

(10) $$\sigma =K(T){\left(\varepsilon_{p}+{\varepsilon_{p}}^{0}\right)}^{n(T)}{\left(\dot{\varepsilon_{p}}\right)}^{m(T)}$$

where σ is the stress; $\varepsilon_{p}$ and $\dot{\varepsilon_{p}}$ are the total plastic strain and the equivalent plastic strain rate ($s^{-1}$), respectively; K(T), n(T), and m(T) are the material constant, the strain hardening coefficient, and the strain rate sensitivity coefficient, respectively, they are temperature-related parameters and the values are obtained from previous publications by Lalpoor [30]; ${\varepsilon_{p}}^{0}$ is the initial strain, and it is set to be 0.001.

Furthermore, we adopted a hot tearing model called HTI [29], which is based on the total strain encountered during solidification, to analyze the effect of different process parameters on the hot tearing susceptibility to the DC process. This model utilized modified Gurson’s constitutive equations to calculate the elastic and plastic strains at a specific node when the solid fraction ranges from 50% to 99%.

#### 2.1.3. Cellular Automaton (CA) Model

The continuous nucleation model proposed by Rappaz was used to simulate the nucleation of grains in the DC casting process [31]. When heterogeneous nucleation occurs, it is distributed across a continuous non-discrete function described by dT/d(∆T).

(11) $$\frac{dT}{d(∆T)}=\frac{n_{max}}{\sqrt{2\pi }∆T_{\sigma }}exp(-\frac{1}{2}{\left(\frac{∆T-∆T_{max}}{∆T_{\sigma }}\right)}^{2})$$

Then the total nucleation density at a certain undercooling ∆T is calculated as

(12) $$n\left(∆T\right)=\int_{0}^{∆T}\frac{dn}{d(∆T)}d(∆T)$$

In the casting process, the total undercooling of the dendrite tip ∆T usually consists of the following four components [26]:

(13) $$∆T=∆T_{c}+∆T_{t}+∆T_{r}+∆T_{k}$$

where $∆T_{c}$, $∆T_{t}$, $∆T_{r}$, and $∆T_{k}$ are the undercooling contributions of solute diffusion, thermal diffusion, solid/liquid interface curvature, and attachment kinetics, respectively.

The Kurz–Giovanola–Trivedi (KGT) [31] model was used to describe the growth rate of columnar crystals and equiaxed crystals during the actual casting. It is considered that $∆T_{c}$ dominates over other factors for most alloys. This model was basically developed for binary alloy systems. Therefore, the equivalent binary approach was adopted to extend this model to the multicomponent Al–Zn–Mg–Cu-based alloy [32], describing the growth rate of dendrite tips as illustrated below:

(14) $$v\left(∆T\right)=a_{2}{∆T}^{2}+a_{3}{∆T}^{3}\;\left\{\begin{array}{c} a_{2}=\frac{2k\Gamma \left(1-k\right)-\rho D^{2}}{2mC_{0}{\left(1-k\right)}^{2}\cdot k\cdot \Gamma }+\frac{mC_{0}}{{D\left(mC_{0}\right)}^{2}(1-k)}\\ a_{3}=\frac{mC_{0}D^{2}}{\pi \Gamma {\left(mC_{0}\right)}^{2}(1-k)\cdot D}\; \end{array}\right.$$

where $a_{2}$ and $a_{3}$ are the dynamic coefficients of dendrite growth; k is the partition coefficient; Γ is the Gibbs–Thomson coefficient ($\Gamma =2\times 10^{-7}$ in this study); m is the slope of the Al-X liquidus; D is the solute diffusion coefficient in the liquid phase.

#### 2.1.4. Multiple Linear Regression Machine Learning Models

**Figure 2:**
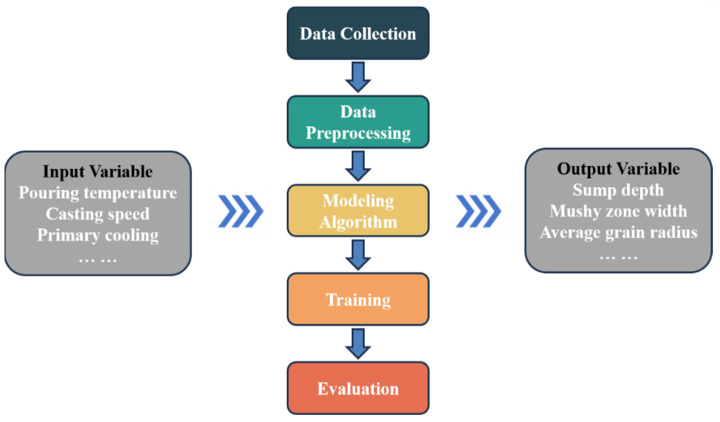
Schematic representation of the machine learning prediction employed in this study.

Figure 2 depicts a schematic representation of the machine learning prediction approach employed in this study. The machine learning was conducted with different process parameters as input variables, and the corresponding mechanical property values of ingots as output variables. The input results were summarized from the finite element simulation and the collected data were normalized to be suitable for machine learning. Then, the machine learning model was trained by substituting the data into a multiple linear regression modeling algorithm.

After preliminary analysis of the simulated data, this study employed machine learning with the multiple linear regression (MLR) algorithm to quantitatively analyze the relationship between important physical parameters such as the sump depth and the mushy zone width in the DC casting process and different process parameters. MLR is a widely used regression algorithm that identifies the linear correlation between a phenomenon influenced by multiple factors and independent variables [33]. It reads as [34]

(15) $$\tilde{y}=\alpha_{0}+\alpha_{1}x_{1}+\alpha_{2}x_{2}+\ldots +\alpha_{n}x_{n}+\delta$$

where $\tilde{y}$ is output (dependent) variable, which is the predictions derived from machine learning analysis. $x_{n}$ denotes input (independent) variables, which in this study, are respectively the value of the pouring temperature, casting speed, PCI, and SCWFR. $\alpha_{n}$ is the coefficient that corresponds to each independent variable, and δ is the error value.

For a multiple regression analysis with m outputs, it is expected that the sum of the residuals (RSS) is as small as possible,

(16) $$RSS=\underset{i=1}{\overset{m}{\sum }}\delta_{i}^{2}=\delta_{1}^{2}+\delta_{2}^{2}+\ldots +\delta_{m}^{2}$$

By performing partial derivatives on the variables in Equation (15) and minimizing the sum of squared residuals (RSS), we can derive the regression coefficient $\alpha_{n}$ and the regression constant $\alpha_{0}$, as shown below:

(17) $$\left\{\begin{array}{c} \frac{\vartheta RSS}{\vartheta \alpha_{0}}=-2\sum_{m=1}^{N}\left[y_{m}-{\tilde{y}}_{m}\right]=0\\ \frac{\vartheta RSS}{\vartheta \alpha_{1}}=-2\sum_{m=1}^{N}\left[y_{m}-{\tilde{y}}_{m}\right]=0\\ \ldots \ldots \\ \frac{\vartheta RSS}{\vartheta \alpha_{n}}=-2\sum_{m=1}^{N}\left[y_{m}-{\tilde{y}}_{m}\right]=0 \end{array}\right.$$

### 2.2. Basic Assumptions of the Model

To simplify the casting process and ensure the reliability of the simulation results, the following simplifications and assumptions were introduced in this study: (I) the melt is a compressible Newtonian fluid with isotropic thermal conductivity; (II) the solidification process satisfies the local thermodynamic equilibrium condition, and the thermal and curvature supercooling is ignored; (III) overlooking the initial DC casting stage, the simulation commences with the instantaneous filling of the cavity formed by the crystallizer and the bottom block, which is instantaneously filled with high-temperature molten aluminum liquid at the same temperature.

### 2.3. Geometry and Boundary Conditions of DC Model

#### 2.3.1. Geometry and Material Properties

Considering the geometric symmetry of the flat ingot structure, 1/4 of the flat ingot (750 mm × 150 mm) was used for analysis. The height of the crystallizer is 190 mm, and the radius of the arc at the corner of the ingot is 68 mm. The ingot remains stationary while the boundary conditions move along the direction opposite to the casting process.

**Figure 3:**
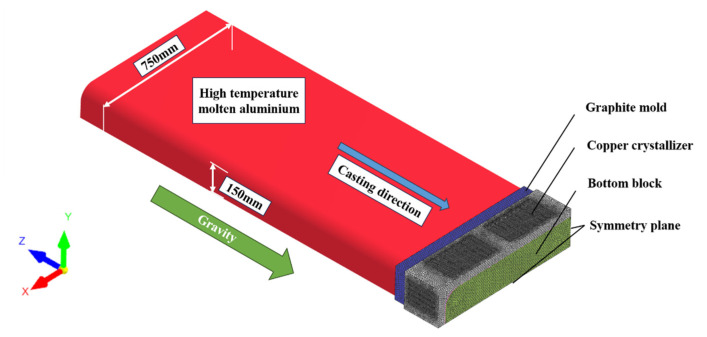
Geometry and grids used for numerical simulations.

Figure 3 depicts the geometry and grid utilized in the numerical simulation, in which the crystallizer section is composed of a water-cooled copper sleeve and a graphite mold. A water-restricted panel is positioned 530 mm below the crystallizer and begins scraping once the casting length reaches 720 mm. The influence of grid size on simulation results was examined (see Figure S1 in Supplementary Materials). Therefore, the uniform grid dimensions of the slab and the crystallizer were set to 10 mm in this study, considering the balance between the accuracy of numerical calculation and computation amount. Furthermore, the grid of the crystallizer surface was refined to enhance heat transfer and accommodate any deformation, and the grid dimensions for the refined surface were reduced to 5 mm. The final model’s count of the triangular grid was 127,497, and the number of tetrahedral elements was 1,232,188.

**Figure 4:**
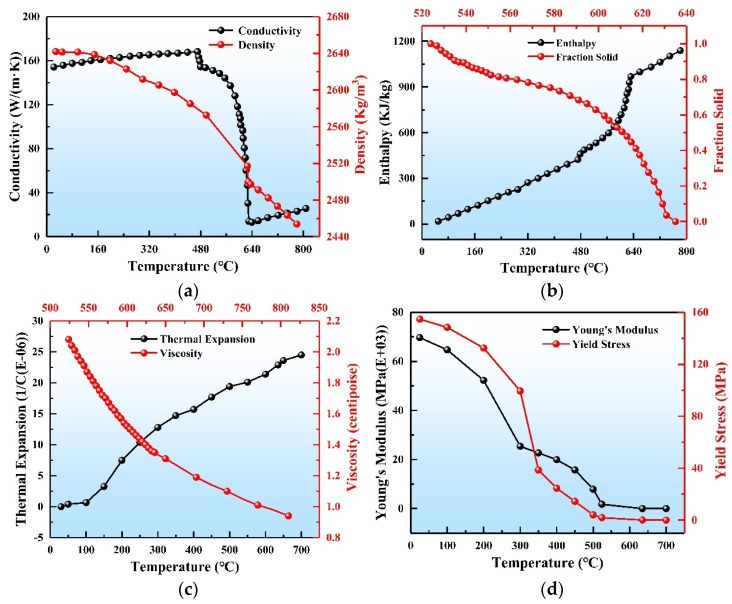
7050 aluminum alloy material properties: (**a**) thermal conductivity and density, (**b**) enthalpy and solid fraction, (**c**) viscosity and thermal expansion coefficient, (**d**) Young’s modulus and yield stress.

In this study, 7050 aluminum alloy was adopted as a representative of the 7xxx series of high-strength aluminum alloys. Its chemical compositions are outlined in Table 1. The liquidus for 7050 aluminum alloy is 635 °C, while the solidus is 524 °C [35,36]. The remaining thermophysical parameters were calculated using JMatPro 7.0 and Thermal Calc 2021b software, and were adjusted using data from tensile experiments. The thermophysical properties used in the final simulation are shown in Figure 4.

#### 2.3.2. Boundary Conditions

**Figure 5:**
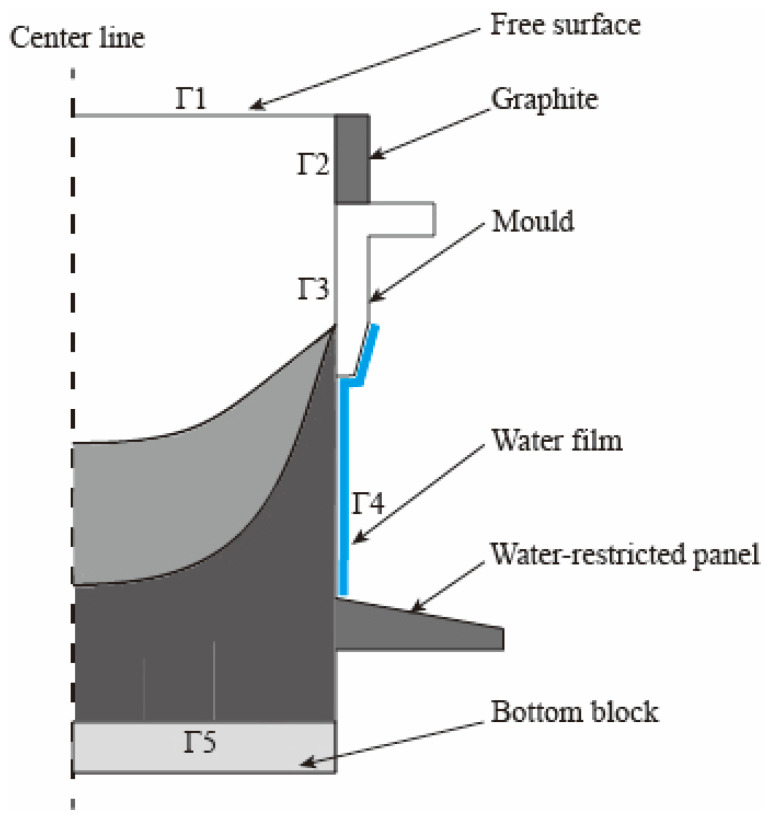
Boundary conditions for the centrosymmetric simulation of DC casting.

**Figure 6:**
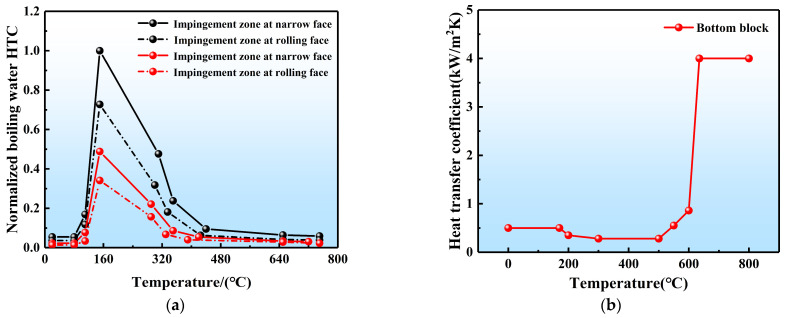
Heat transfer coefficient used in the simulation: (**a**) secondary cooling boundary, (**b**) bottom cooling boundary.

The boundary conditions for the simulations were introduced as shown in Figure 5 and Table 2. Based on the Weckman–Niessen empirical formula [37] and the results of Drezet et al. [38], the effect of varying SCWFRs on the DC casting process was simulated in this study by multiplying the nominalized boiling curves by different scale factors. The nominalized boiling curves are shown in Figure 6, and the scale factors were calculated using the peak value at the boiling heat exchange point according to the Weckman–Niessen empirical formula [1,4,11]. The impact of the water-restricted panel on the DC casting process was evaluated by treating the area beneath the water scraper as an air-cooled section at the secondary cooling boundary. The impact of the water-restricted panel on the DC process was evaluated by treating the area beneath the water limiter as an air-cooled section at the secondary cooling boundary. Radiative heat loss was ignored for simplicity.

### 2.4. Numerical Implementation

The commercial finite element ProCAST 2021 software was employed to facilitate the numerical simulation of the DC casting process for large-scale flat ingots of 7050 aluminum alloy. In this study, the influences of various pouring temperatures, casting speeds, PCIs, and SCWFRs on the DC casting process were examined using the simulation parameters outlined in Section 2.3.

A total of 102 simulations were performed, and divided by 4 groups as shown in Table 3. As shown in the table, simulation cases within the same group only differ in individual process parameters. The table also shows the variations in range and step size. (The results for a first-cooling intensity of 3000 W/(m^2^·k) versus a second-cooling water flow rate of 420 L/min are not presented in Section 3.3 and Section 3.4 for graphical enhancement. They are only discussed in the context of machine learning.)

After acquiring the calculated data from the simulation, we used the hold-out method to divide the dataset into training and validation sets. To facilitate the analysis, maximum–minimum normalization was applied to the data. Finally, we carried out an MLR analysis by using Anaconda 3 and pymatgen 7 [41].

## 3. Results and Discussion

### 3.1. Validation of DC Casting Model

Yu et al. [35] have conducted temperature and sump profile measurements of 7050 round DC cast ingots with a 300 mm diameter by using thermocouples and pouring Al-30%Cu melt. In order to verify the reliability of the results in this paper, we compared our simulation results with previous experimental measurements and computational results in terms of the temperature distribution and sump profiles.

**Figure 7:**
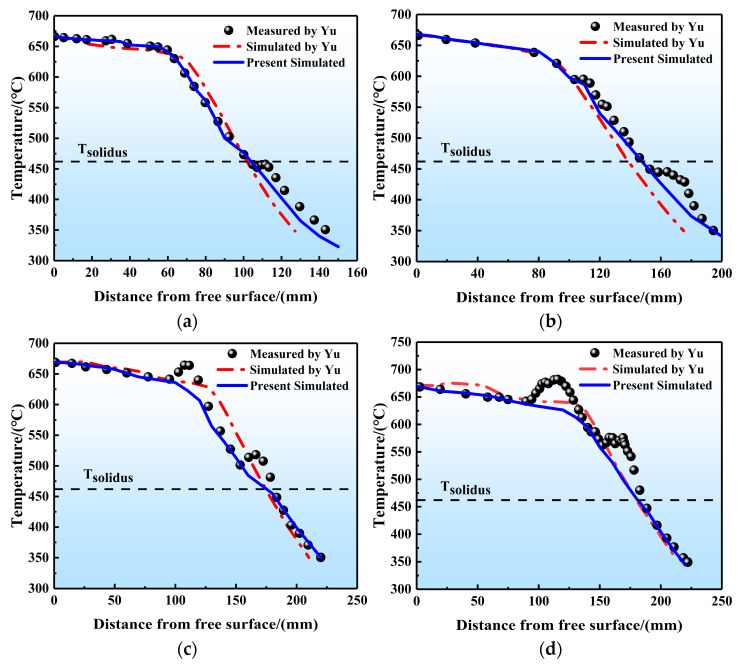
Temperatures profiles as a function of the distance from free surface obtained by numerical simulation and experimental measurement for the round ingots with different radius R: (**a**) R = 0.015 m, (**b**) R = 0.045 m, (**c**) R = 0.12 m, (**d**) R = 0.15 m [35].

**Figure 8:**
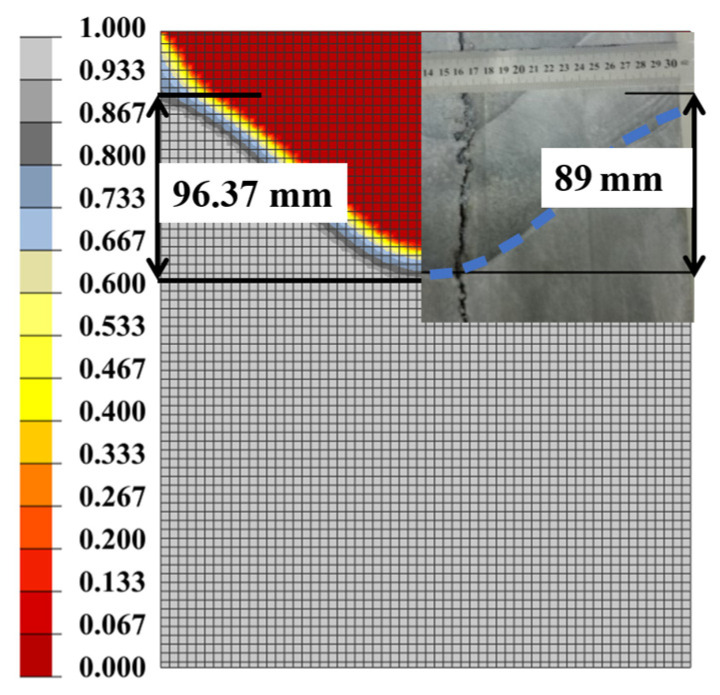
Calculated and experimental sump profiles (the result of the sump profile on the right side of the figure is the experimental results traced by Yu [35]).

Figure 7 shows the vertical temperature profile of round ingots at four different radii (0.015 m, 0.045 m, 0.12 m, and 0.15 m). It is evident that the shape and value of the temperature curves obtained by our simulations agree well with those obtained by Yu’s study. As shown in Figure 7, the high-temperature aluminum liquid experiences gradual cooling due to the crystallizer, causing a relatively slow decrease in temperature with the distance from the bulk of melt to surface. Upon entering the secondary cooling zone and making contact with the cooling water, the temperature drops rapidly from about 630 °C to 350 °C. Moreover, as shown in Figure 8, we compared quantitatively the sump profiles of round ingots at steady state obtained in experiments and our simulation. The results show excellent agreement between the measured and calculated profiles. Additionally, the predicted sump depth is 96.37 mm, which closely matches the actual depth of 89 mm. Therefore, the good agreements of temperature distribution and solid–liquid interface (sump) shape demonstrate the reasonability of our simulations.

### 3.2. Effect of Pouring Temperature in DC Casting

**Figure 9:**
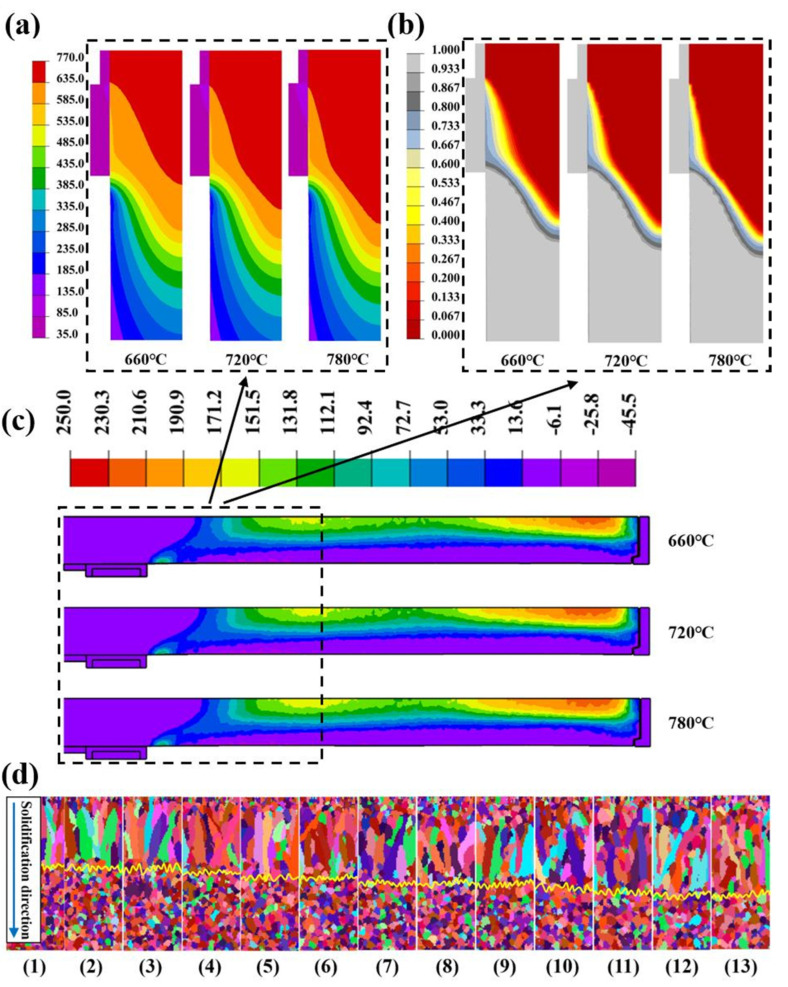
Contour maps of different pouring temperatures on DC casting: (**a**) temperature field, (**b**) solid fraction, (**c**) stress field, (**d**) solidified structure, numbers 1–13 represents the casting temperatures for the different counts in Group A of Table 3, in the range 660~780 °C.

**Figure 10:**
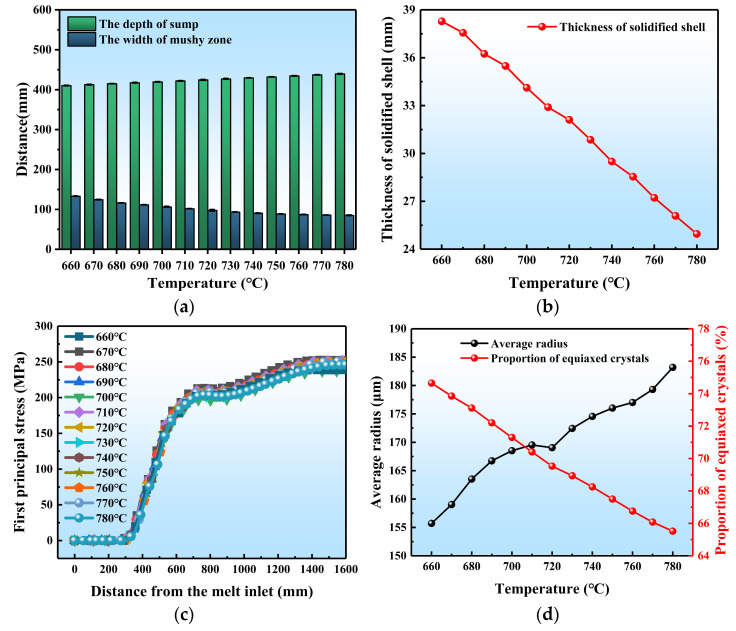
Quantitative results of different pouring temperatures on DC casting: (**a**) the sump depth and mushy zone width, (**b**) the initial solidified shell thickness, (**c**) the first principal stress, (**d**) the average grain radius and proportion of equiaxed crystals.

Then, as shown in Figure 9a and Figure 10a, we investigated the influence of pouring temperature on the DC casting process of large-scale 7050 aluminum alloy flat ingots. Figures S6 and S7 in Supplementary Materials display the locations of the first principal stress and microstructure measurement points, respectively. As pouring temperature increases, the isotherm shifts downward, the sump depth increases from 410.35 mm at 660 °C to 439.65 mm at 780 °C, and the mushy zone width decreases from 133.23 mm to 85.22 mm. For every 10 °C increase in temperature, the initial solidified shell thickness decreases by about 1.2 mm. Stress was not sensitive to casting temperature, decreasing slightly from 252.5 MPa to 245.8 MPa. The ingot still maintains a tensile stress in the core, and a compressive stress in the surface layer. This is due to the fact that the change in the heat capacity of the melt caused by changing the pouring temperature only accounts for a very small portion of the total heat input, the overall temperature field of the ingot remains relatively stable under constant conditions. However, the temperature gradient has a significant impact on internal thermal stress. Thus, a small change in the temperature gradient ultimately leads to insignificant changes in the final thermal stress.

As shown in Figure 9d and Figure 10d, the average grain radius of the ingot increases from 155.7 μm to 183.2 μm with increasing temperature, while the proportion of equiaxed crystals decreases from 74.65% to 65.52%. It means that the microstructure of the DC casting ingot at a low casting temperature is finer and more uniform. From the perspective of nucleation kinetics, the lower the casting temperature, the more likely it is to reach the undercooling required by the nucleation in the center of the ingot. This is conducive to nucleation, resulting in a profound increase in nuclei. Consequently, the grains were substantially refined. Meanwhile, as the pouring temperature decreases, the width of the mushy zone increases, and the temperature gradient at the solidification front consequently decreases. When this decrease is so small that the undercooling of the solidification front exceeds the undercooling required by nucleation, the nucleus would be nucleated at the solid–liquid interface and grow rapidly. This prevents the columnar crystals from continuously growing into the interior of the ingot, resulting in the successful transition from columnar to equiaxial crystal structure (CET) and the increased proportion of equiaxed crystal.

### 3.3. Effect of Casting Speed in DC Casting

**Figure 11:**
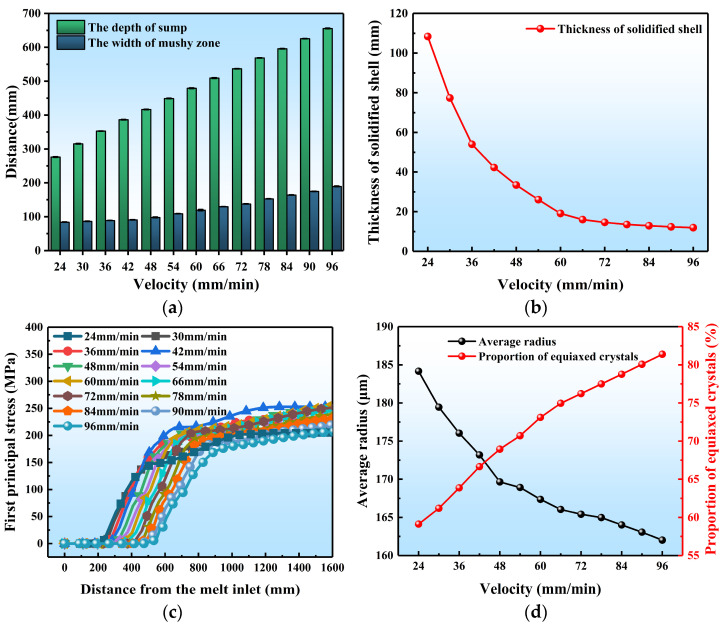
Quantitative results of different casting velocities on DC casting: (**a**) the sump depth and mushy zone width, (**b**) the initial solidified shell thickness, (**c**) the first principal stress, (**d**) the average grain radius and proportion of equiaxed crystals.

Casting speed is one of the most crucial process parameters of DC casting. In this section, we studied the changes in each physical field during DC casting at different casting velocities. The quantitative description is shown in Figure 11, and the contour maps are shown in Figure S1 in Supplementary Materials.

As shown in Figure 11a, the depth of the sump increases from 276.28 mm at a casting speed of 24 mm/min to 655.67 mm at a speed of 96 mm/min. Accordingly, the width of the mushy zone increases from 84.02 mm to 189.51 mm. This is because the residence time for the ingot in various cooling regions decreases as the speed increases, thereby reducing the cooling intensity of the ingot. Moreover, Figure 11b shows that the initial solidified shell thickness decreases rapidly with increasing casting speed. The thickness decreases steeply from 108.4 mm to 19.1 mm as casting speed increases from 24 mm/min to 60 mm/min, then the decreasing rate slows down gradually. Consequently, the trend of decreasing solidified shell thickness continues to decrease, resulting in a final reduction from 19.1 mm to 11.97 mm. If the casting speed is higher than 70 mm/min, serious liquid leakage would occur.

As shown in Figure 11c, the increase in speed significantly reduces the first principal stress within the ingot. The maximum stress is 257.2 MPa at a speed of 60 mm/min, and it decreases to 206.1 MPa at a speed of 96 mm/min. This differs significantly from the results of Lalpoor et al. [6]. It has been reported that the first principal stress increases notably with the casting speed of round ingots. This might be related to the differences in the distribution of stress components between round and billet ingots. In round ingots, the peak stress components are typically located at the center of the ingot, whereas in flat ingots, the width of the rolled side of the ingot is several (usually 2.5–5) times wider than that of the narrow side, which leads to an inconsistent distribution of peak stress components. When the casting speed changes, it would lead to changes in the distribution of various stress components, ultimately causing substantial changes in the first principal stress within the ingot.

In addition, as shown in Figure 11d, the average grain radius in the ingot decreases from 184.16 μm to 162.02 μm with the casting speed, and the decreasing trend gradually slows down. Meanwhile, the proportion of equiaxed crystals increases from 59.12% to 81.39%. This is because the increase in speed leads to a decrease in the temperature gradient of the ingot’s mushy zone, slowing down the solidification rate of the billet and leading to a more uniform temperature distribution. This, in turn, results in a finer and more uniform structure of the ingot at high casting speeds, which is in line with the practices of DC casting in reality.

### 3.4. Effect of PCI in DC Casting

Although the heat lost during the primary cooling stage only represents 20% of the overall cooling phase of the ingot, it is crucial to the final surface quality of the ingot and the occurrence of casting defects such as cold shut and liquid leakage. This subsection examined the impact of PCIs on the DC casting process.

**Figure 12:**
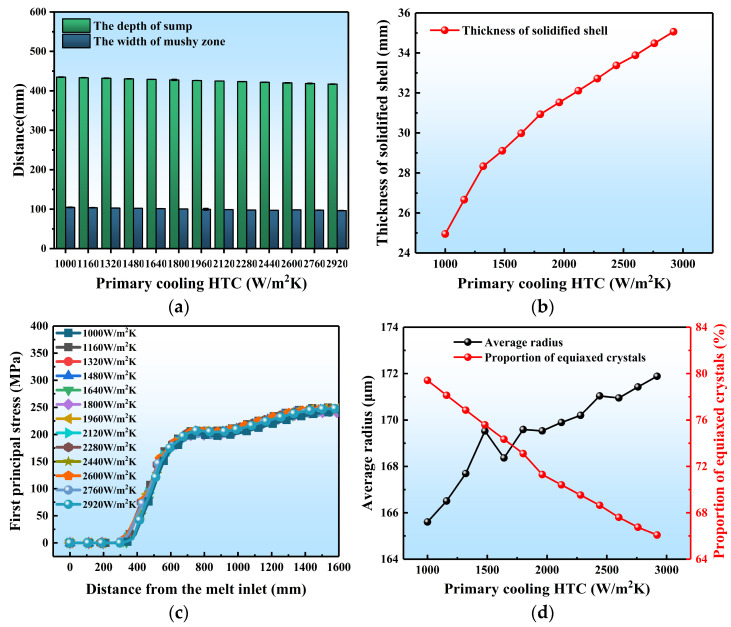
Quantitative results of different PCIs on DC casting: (**a**) the sump depth and mushy zone width, (**b**) the initial solidified shell thickness, (**c**) the first principal stress, (**d**) the average grain radius and proportion of equiaxed crystals.

Generally, the quantitative analysis in Figure 12 and the contour maps in Figure S2 in Supplementary Materials indicate that the depth of the sump, the width of the mushy zone, and the stress in the ingot are not significantly affected by the intensity of the primary cooling. However, as shown in Figure 12b, the thickness of the initial solidified shell increases from 24.95 mm to 35.06 mm as the cooling intensity increases from 1000 to 2920 W/(m^2^·k). Once the cooling intensity reaches a certain value (2000 W/(m^2^·k) in this article), the increasing trend of the thickness of the solidified shell gradually slows down. This is because increasing the PCI does not cause noteworthy alterations in the temperature gradient of the ingot. The intensity primarily facilitates the convective heat transfer of the melt in the mold, leading to quick cooling of the local melt in touch with the mold. As a result, the initial solidified shell thickness increases. Since the heat transfer process is also influenced by other factors including the external ambient temperature and the materials used for the mold, the increase in PCI may not result in an obvious change.

As shown in Figure 12d, with the PCI increasing from 1000 to 2920 W/m^2^·k, the average grain radius of the ingot gradually increases from 165.6 μm to 171.9 μm, while the proportion of equiaxed crystals decreases from 79.42% to 66.08%. This is because increasing the PCI accelerates the transition of the chill zone to columnar crystals ultimately promotes the growth of these crystals. Generally, the influence of the primary cooling intensity on solidification structure is very limited.

### 3.5. Effect of SCWFR in DC Casting

**Figure 13:**
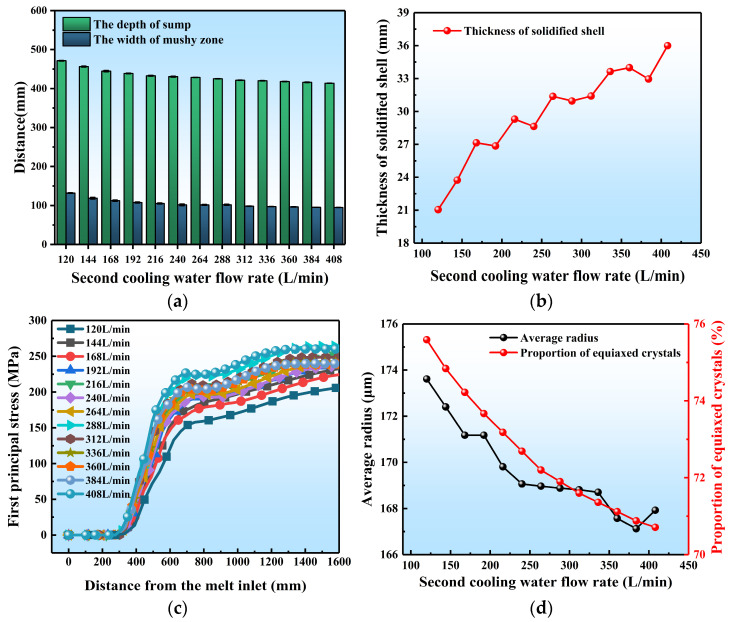
Quantitative results of different SCWLRs on DC casting: (**a**) the sump depth and mushy zone width, (**b**) the initial solidified shell thickness, (**c**) the first principal stress, (**d**) the average grain radius and proportion of equiaxed crystals.

Figure 13 presents the variation of various physical properties during DC casting under different SCWFRs. The corresponding contour maps are given in Figure S3 in Supplementary Materials. With the water flow rate increasing from 120 to 408 L/min, the depth of the sump and the width of the mushy zone in the ingot both decrease, from 471.41 mm and 210.50 mm at 120 L/min, to 413.71 mm and 94.90 mm at 408 L/min, respectively. Meanwhile, the initial solidified shell thickness gradually increases from 21.05 mm to 33.99 mm. The increase in the SCWLR leads to a shallower sump, which reduces the temperature difference between the inside and outside of the crystallizer. This is equivalent to a decrease in pouring temperature. Consequently, there is a gradual increase in the initial solidified shell thickness.

As shown in Figure 13c, the increase in water flow rate significantly increases the first principal stress in the ingot, from 205.9 MPa to 260.1 MPa. This increase is attributed to the rise in temperature gradient within the ingot. The average grain radius decreases from 173.6 μm to 167.9 μm as the water flow rate increases. An increase in the SCWLR causes a greater temperature lag during the nucleation process of solidification, resulting in a higher degree of undercooling. This increase in undercooling reduces the critical radius for nucleation, leading to a higher nucleation rate. Additionally, rapid cooling reduces the diffusion and mobility of atoms, resulting in numerous crystal nucleus that form but do not have sufficient time to grow fully, remaining small in size. Meanwhile, the proportion of equiaxed crystals decreases from 75.59% to 70.71% accordingly.

Furthermore, it is important to note that once the water flow rate reaches a certain value, its impact on the ingot gradually decreases. This is due to the fact that the heat transfer process between the ingot and the external environment is not solely dependent on the heat transfer between the ingot and the external surroundings, but also involves the heat dissipation process of the ingot itself through conduction to the outside environment. When the heat transfer process dominates over the heat conduction process of the ingot itself, a further increase in the water flow rate has a negligible impact on improving the heat transfer coefficient. At this point, the thermal conductivity of the ingot becomes the limiting factor for heat dissipation.

### 3.6. Multiple Linear Regression Analysis

The results of the ProCAST simulations demonstrate that the influences of multiple casting parameters on the temperature field, stress field, and microstructure of the ingot are extremely intricate. Precise control of the casting recipe is challenging due to reasons such as different weights, offset effects, and non-linearity among the influences of casting parameters. Therefore, establishing the input–output relationship for aluminum alloy in the DC casting process remains a formidable challenge. Numerous studies conducted recently demonstrate that machine learning possesses high accuracy and efficacy in predicting multivariate problems. Hence, MLR is used in this section to predict the quality of the DC cast ingot under different casting processes, based on the physical quantities calculated above (the quantitative analysis is given in Figure S4 in Supplementary Materials). Considering the vast gap about the magnitudes of different physical quantities in the original dataset, the dataset has been normalized by using the min–max normalization. The predicting model was obtained through MLR analysis of the above ProCAST simulation data; it can be described as follows,

(18) $$y_{sump}=0.077196x_{1}+0.96872x_{2}-0.04812x_{3}-0.12467x_{4}+0.093416$$

(19) $$y_{mushy}=-0.43883x_{1}+1.1219x_{2}-0.07831x_{3}-0.26575x_{4}+0.16964$$

(20) $$y_{shell}=-0.1406x_{1}-0.6796x_{2}+0.09806x_{3}+0.124x_{4}+0.43126$$

(21) $$y_{HCS}=0.27734x_{1}-0.25606x_{2}-0.05516x_{3}+0.8868x_{4}+0.14565$$

(22) $$y_{proportion}=-0.42532x_{1}+0.8787x_{2}-0.63x_{3}-0.216x_{4}+0.846$$

(23) $$y_{radius}=0.8327x_{1}-0.59465x_{2}+0.18425x_{3}-0.1982x_{4}+0.31895$$

**Figure 14:**
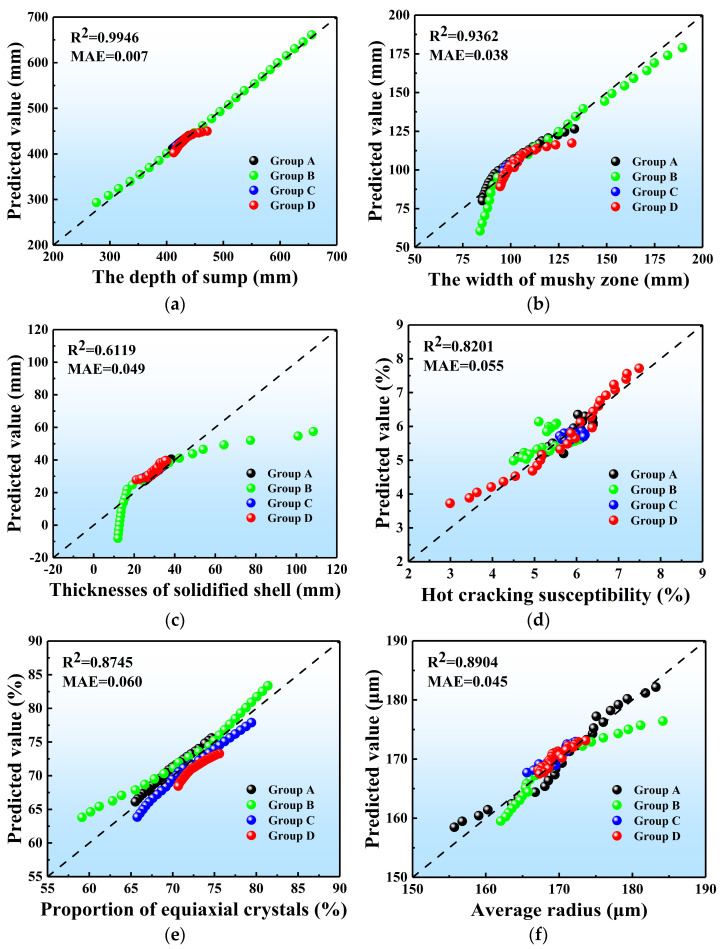
Predictive values of MLR machine learning models on the simulated dataset: (**a**) the depth of sump, (**b**) the width of mushy zone, (**c**) the thickness of the initial solidified shell, (**d**) the hot cracking susceptibility, (**e**) the proportion of equiaxed crystals, (**f**) the average grain radius.

Figure 14 displays a comparison between the predicted values and the results obtained from the simulation, where Group A, B, C, and D represent the different pouring temperatures, casting velocities, PCIs, and SCWFRs, respectively. In this study, we utilized the coefficient of determination $R^{2}$ and mean absolute error (MAE) as evaluation metrics for gauging the correlation between two variables. *R*^2^ is an evaluation parameter that reflects the proximity between the true value and the fitted regression value. It is a unitless measure that ranges from 0 to 1 and reflects the relative magnitude of the regression contribution, indicating the percentage of the total variation in the dependent variable *Y* that is explained by the regression relationship. And MAE is the average of the absolute deviations of all individual regression value from the arithmetic mean value. It reflects the error in the prediction value. They are calculated as

(24) $$R^{2}=1-\frac{\mu }{\nu }=1-\frac{\sum {\left(y_{i}-\tilde{y_{i}}\right)}^{2}}{\sum {\left(y_{i}-{\hat{y}}_{i}\right)}^{2}}$$

(25) $$MAE=\frac{1}{n}\underset{i=1}{\overset{n}{\sum }}|(y_{i}-\tilde{y_{i}})|$$

where *μ* and *ν* are the residual sum of squares, the total sum of squares, respectively.

A larger deviation between the actual and projected values results in a smaller *R*^2^ value and a larger MAE value. The accuracy of the prediction is evaluated using the *R*^2^ and MAE values. Generally, the results with an *R*^2^ value above 0.6 [42] and a small MAE are acceptable. From Figure 14, the *R*^2^ and MAE values for the sump depth, mushy zone width, initial solidified shell thickness, first principal stress, proportion of equiaxed crystals, and average grain radius are (0.9946, 0.007), (0.9362, 0.038), (0.6119, 0.049), (0.8201, 0.055), (0.8745, 0.060), and (0.8904, 0.045), respectively, suggesting excellent agreement between the predicted and simulated results.

All of the above predictions are based on data obtained from our numerical simulations. To evaluate the accuracy of these predictions in predicting the quality of actual DC casting ingots, the experimental results obtained by Drezet, Wan J, Subodh, etc. [43,44,45,46,47] are used to compare with the predicted results of the predicting model in this study. Given the difficulty in obtaining accurate stress results during the casting process, the literature typically assesses the magnitude of stresses in ingots through hot tearing susceptibility [28]. Therefore, only the temperature field (the sump depth) and microstructure (the average grain radius) are available in these studies and could be used to evaluate the predictive performance of the model.

After normalizing the casting conditions according to the boundary conditions stipulated in the model, the experimental data collected are used to calculate the predicted sump depth through Equation (18) and average grain radius through Equation (23). The comparison between the predicted and actual results is outlined in Table 4.

It is evident that the depths of the sump in the ingots predicted by this model generally agree very well with the experimental results. For flat ingots, the discrepancy between the predicted and actual results is negligible, reaching a maximum of only 7.39%. However, the model has limitations in predicting the average grain radius of the ingot. When the size of ingot was relatively small, the error could be as high as 57.6%. This is because the smaller the ingot size, the faster the cooling rate, which is more favorable for nucleation and the eventual formation of a fine-grained structure. Furthermore, the solidification rate varies at different locations in the ingot due to changes in process parameters and casting stages during the casting process. The parameters for nucleation, such as the maximum density of nucleation and the undercooling required for nucleation in molten metal, are dependent on the cooling rate for a specific type of heterogeneous nucleation core. Therefore, the nucleation parameters are not constant within the ingot. Due to the limited experimental conditions and the scarcity of the literature reports on nucleation parameters for continuous casting processes, we can only refer to the nucleation parameters under the relevant casting process and treat them as constants in this manuscript. As a result, the model’s prediction error about grain size is significant.

Generally, this model demonstrates satisfactory performance in predicting the quality of DC cast ingots under various casting processes. By comparing the predicted results with the desired results, foundry engineers can accelerate the acquisition of optimal casting recipe. Experienced engineers can even reduce casting defects based on these predictions. However, its specific applicability is constrained by factors such as the geometry of the ingot and accuracy of material physical parameters. This is because the process of producing ingots is impacted not only by process parameters but also by several factors, including the physical and chemical properties of alloys, the quality of the cooling water, the structure of crystallizer, and the water impact angle. Errors may arise when these factors are not taken into account. In addition, highly accurate material physical property parameters are essential for improving the prediction accuracy. To broaden the future application of the prediction model, it is imperative to gather increasingly precise data of DC casting production to consider a wider range of influential factors and thereby reduce errors. Furthermore, it is worth considering additional physical variables related to the DC casting process, including the concentration field and defect density.

## 4. Conclusions

This paper presents a comprehensive study on the evolution of multi-physical fields and grain structures during DC casting solidification processes, providing a reference and method for the optimization of DC casting processes for large-size aluminum alloy flat ingots. Based on the results and discussions, the following conclusions can be drawn:

1. The finite element model used in this manuscript underwent validation against the temperature and sump profiles measured by Yu et al. [35]. The findings indicated that the model used in this study agrees well with the experimental results.
2. Through the analysis of process optimization, we quantitatively analyzed how multiple process parameters (such as pouring temperature, casting speed, and cooling conditions) affect the DC casting process. The results show that the influences of casting parameters on DC casting processes are very complex, exhibiting characteristics such as different weight coefficients, additive or offsetting, non-linearity, and so on.
3. A novel and efficient method to predict the DC casting process was proposed. By using the integrated computational method combining numerical simulations with machine learning, a novel predicting model about correlation between the process of aluminum alloy DC casting ingots and the casting recipe was established. This model could accurately predict the quality of DC casting ingots, and show good consistency with experimental results by reasonably taking into account the geometry of ingot and material properties of alloys.

## Figures and Tables

**Table 1:** Nominal composition of 7050 Aluminum Alloy (wt%).

Element	Zn	Cu	Mg	Fe	Cr	Mn	Si	Zr	Ti	Al
Content(wt%)	6.0	2.15	2.3	0.10	0.04	0.10	0.03	0.11	0.03	Bal.

**Table 2:** Boundary conditions for the faces depicted in Figure 5.

Boundary Conditions	
***Pressure P***	
Free surface Γ1	Dirichlet boundary condition with a fixed value of 1 × 10^5^ Pa
***Temperature T***	
Free surface Γ1	Dirichlet boundary condition, value equal to the casting temperature
Graphite Γ2	Heat transfer coefficient equal to 100 W/(m^2^∙K)
Mould Γ3	Calculated by $h=h_{con}\times \left(1-f_{s}\right)+{h_{air}\times f}_{s}$ [1,3]
Water film Γ4	As shown in Figure 6a
Bottom block Γ5	As shown in Figure 6b [39,40]
	External temperature set to 25 °C The remaining heat exchange interfaces are set to adiabatic
***Velocity V***	
Bottom block	Dirichlet boundary condition, value equal to the casting speed
***Nucleation N***	
Ingot	$a_{2}=1.667\times 10^{-7}$, $a_{3}=2.810\times 10^{-8}$ [36]
Surface nucleation	$∆{\bar{T}}_{S}=1\;K$, $∆T_{\sigma ,S}=0.1\;K$, $n_{S,max}=9.15\times 10^{5}\;m^{-2}$
Volume nucleation	$∆{\bar{T}}_{V}=20\;K$, $∆T_{\sigma ,V}=1\;K$, $n_{V,max}=7.0\times 10^{7}\;m^{-3}$

**Table 3:** Simulation cases in this study.

Gp.	Temperature [°C]	Speed [mm/min]	PCI [W·m^−2^·K^−1^]	SCWFR [L/min]
A	660~780 ΔT = 5	50	2000	270
B	720	24~96 ΔV = 3	2000	270
C	720	50	1000~3000 ΔPCI = 80	270
D	720	50	2000	120~420 ΔWFR = 12

**Table 4:** Comparison of predicted and experimental results.

No.	Type of Alloy	Ingot Size [mm]	Experimental Results	Predicted Results	Errors(%)
***The depth of sump* [mm]:**					
1 [43]	AA 1xxx	1860 × 510	425 480 580	419.85 470.93 573.11	1.21 1.89 1.19
2 [44]	AA 7050	1372 × 406	381	406.91	6.8
3 [45]	AA 3104	1320 × 660	440	448.98	2.04
4 [46]	AA 3004	2044 × 680	435	467.17	7.39
***The average grain size* [μm]:**					
1 [46]	AA 3004	2044 × 680	400	321.395	19.65
2 [47]	AA 2524	350 × 160	210 ± 39	330.961	57.6

## Data Availability

Data are contained within the article.

## Supplementary Materials

The following supporting information can be downloaded at: https://www.mdpi.com/article/10.3390/ma17061409/s1, Figure S1: The quantification of the effect of grid size on the simulation results for DC casting; Figure S2: Contour maps of different casting velocities on DC casting; Figure S3: Contour maps of different PCIs on DC casting; Figure S4: Contour maps of different SCWFRs on DC casting; Figure S5: Quantitative results of different process parameters on HCS for DC casting; Figure S6: Schematic of the location for measuring first principal stress; Figure S7: Schematic of the locations for measuring microstructure.

## Abbreviations

**Symbols**	**Nomenclature**
${C_{0}}^{s}$	The reference concentration value for species s
D	Solute diffusion coefficient in the liquid phase
E	Elastic modulus using multiple regression analysis to predict [MPa]
H	Enthalpy [$kJ\cdot {kg}^{-1}$]
K	Heat transfer coefficient [$W\cdot m^{-1}\cdot {{}^{\circ}C}^{-1}$], the material Constant
$K_{P}$	The permeability coefficient
L	Latent heat of solidification [$J\cdot {kg}^{-1}$]
R	Radius of a dendrite tip, billet
$T,T_{water},T_{saturation}$	Temperature, Initial, Saturation temperature of cooling water [°C]
Y	Direction of pull
$a_{2},a_{3}$	Dynamic coefficients of dendrite growth
c	Specific heat [J$\cdot {kg}^{-1}\cdot {{}^{\circ}C}^{-1}$]
${f,\;f}_{s},{f_{s}}^{th}$	Yield stress, solid fraction, packing limit
$h_{con}$	Heat transfer coefficient of the melt contact to the mold [$W\cdot m^{-2}\cdot K^{-1}$]
$h_{air}$	Heat transfer coefficient at air gap [$W\cdot m^{-2}\cdot K^{-1}$], $h_{air}=150\;[\;W\cdot m^{-2}\cdot K^{-1}]$
k	The partition coefficient
m	The slope of the Al-X liquidus, the strain rate sensitivity Coefficient
n	Nucleation density [$m^{-3}$], the strain hardening coefficient
$n_{max}$	Maximum nucleation density [$m^{-3}$]
$y,\tilde{y},{\hat{y}}_{i}$	Truth value, predicted value, the overall mean of truth value
ρ	Density [$kg\cdot m^{-3}$]
p	Pressure [Pa]
t	Time [s]
u	Velocity [m/s]
$\mu ,\mu_{t}$	The dynamic viscosity, turbulent viscosity
$\alpha_{0},\alpha_{1},\alpha_{2}\ldots \alpha_{n}$	Regression coefficient, $\alpha_{0}=constant$
σ	Node stress [MPa]
${\varepsilon_{s}}^{T},{\varepsilon_{s}}^{\varepsilon },{\varepsilon_{s}}^{p}$	Thermal, elastic, plastic strain
$\varepsilon_{p},{\dot{\varepsilon }}_{p},{\varepsilon_{p}}^{0}$	Total plastic strain, equivalent plastic strain rate [$s^{-1}$], initial strain
δ	Regression errors
μ,ν	Residual sum of squares, total sum of squares
∆T	Total undercooling [°C]
$∆T_{max}$	Mean nucleation undercooling [°C]
$∆T_{\sigma }$	Standard deviation of nucleation undercooling [°C]
$∆T_{c}$	The undercooling contributions of solute diffusion [°C]
$∆T_{t}$	The undercooling contributions of thermal diffusion [°C]
$∆T_{r}$	The undercooling contributions of solid/liquid interface curvature [°C]
$∆T_{k}$	The undercooling contributions of attachment kinetics [°C]
$∆{\bar{T}}_{S},∆{\bar{T}}_{V}$	Surface, bulk nuclear undercooling [K]
$∆T_{\sigma ,S},∆T_{\sigma ,V}$	Standard deviation of surface, bulk nuclear [K]
$n_{S,max},n_{V,max}$	Surface, bulk grain density [m^−2^]
Γ	Gibbs–Thomson coefficient ($\Gamma =2\times 10^{-7}$ in this study)
γ	The plastic multiplier
$\beta_{T},\beta_{S}$	The thermal expansion coefficient, the solute expansion coefficient for species s
