# Supplementary material for: Numerical Simulation and Machine Learning Prediction of the Direct Chill Casting Process of Large-Scale Aluminum Ingots
Source: Materials (Basel). 2024 Mar 19;17(6):1409. doi: 10.3390/ma17061409 (PMC10971882; doi:10.3390/ma17061409)
Supplement: Supplementary file 1 [file materials-17-01409-s001.zip › materials-2886408-supplementary.pdf]

Figure S1 shows the quantification of the effect of grid size on the simulation results for DC casting. From the picture, we can see that the simulation results are minimally affected by the mesh size. Therefore, the uniform grid dimensions of the slab and the crystallizer were set to 10 mm in this study, with computational efficiency and accuracy in mind.

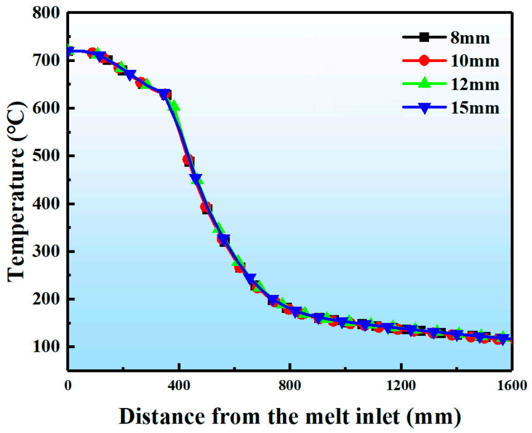

(a)

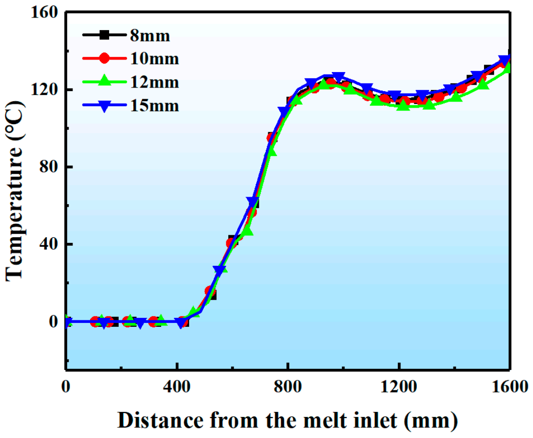

(b)

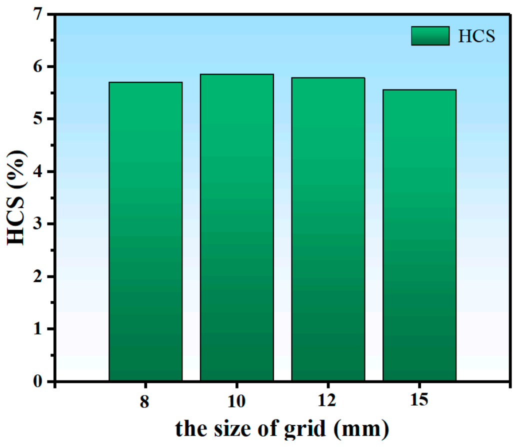

(C)

Figure S1. The quantification of the effect of grid size on the simulation results for DC casting: (a) temperature distribution, (b) strss distribution, (c) hot cracking susceptibility

Figure S2 shows the contour maps results regarding the impact of different casting speed on the temperature field, stress field, and microstructure of the DC cast billet.

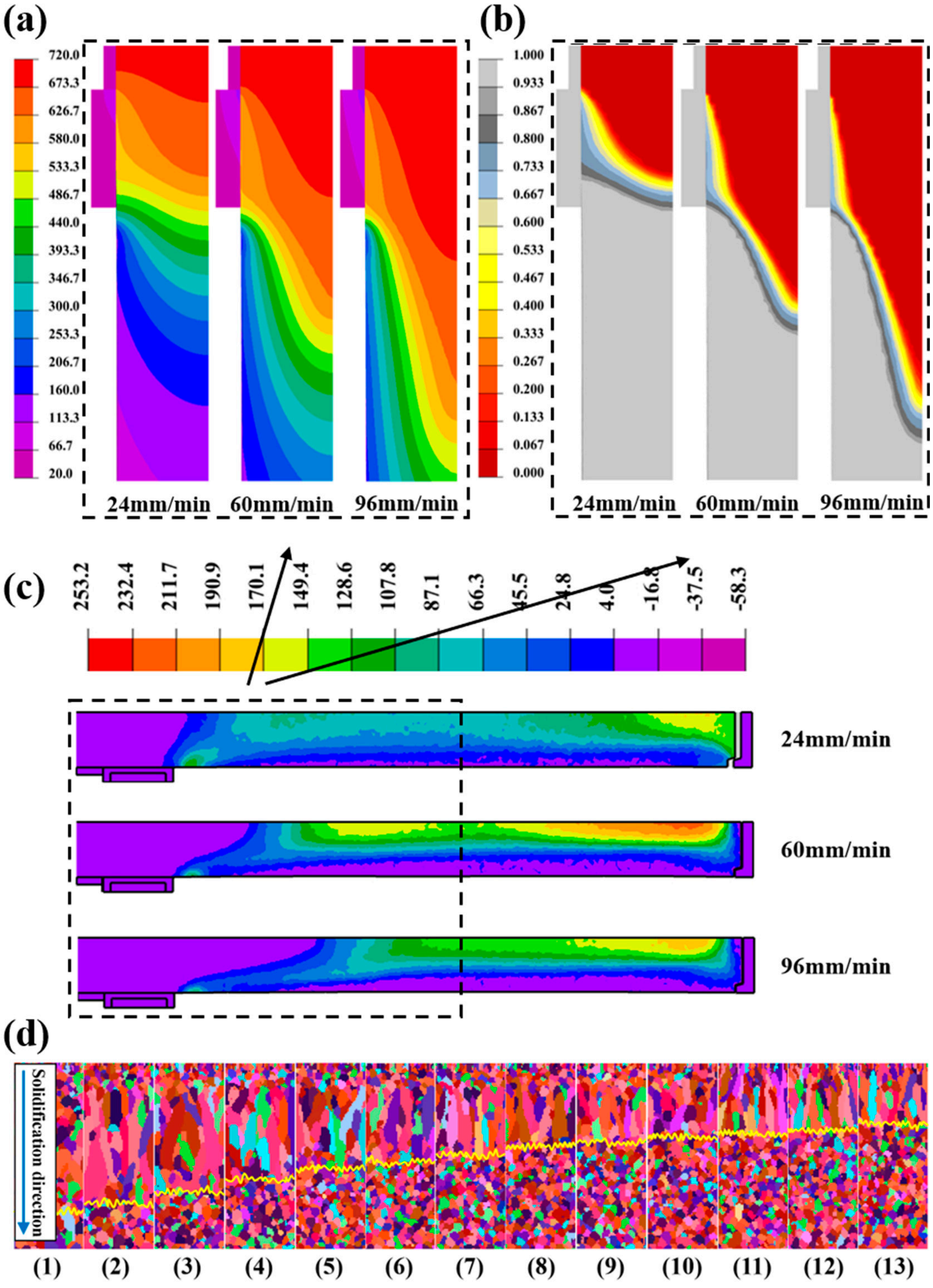

Figure S2. Contour maps of different casting velocities on DC casting: (a) temperature field, (b) solid fraction, (c) stress field, (d) solidified structure.

Figure S3 shows the contour maps results regarding the impact of different primary cooling intensities on the temperature field, stress field, and microstructure of the DC cast billet.

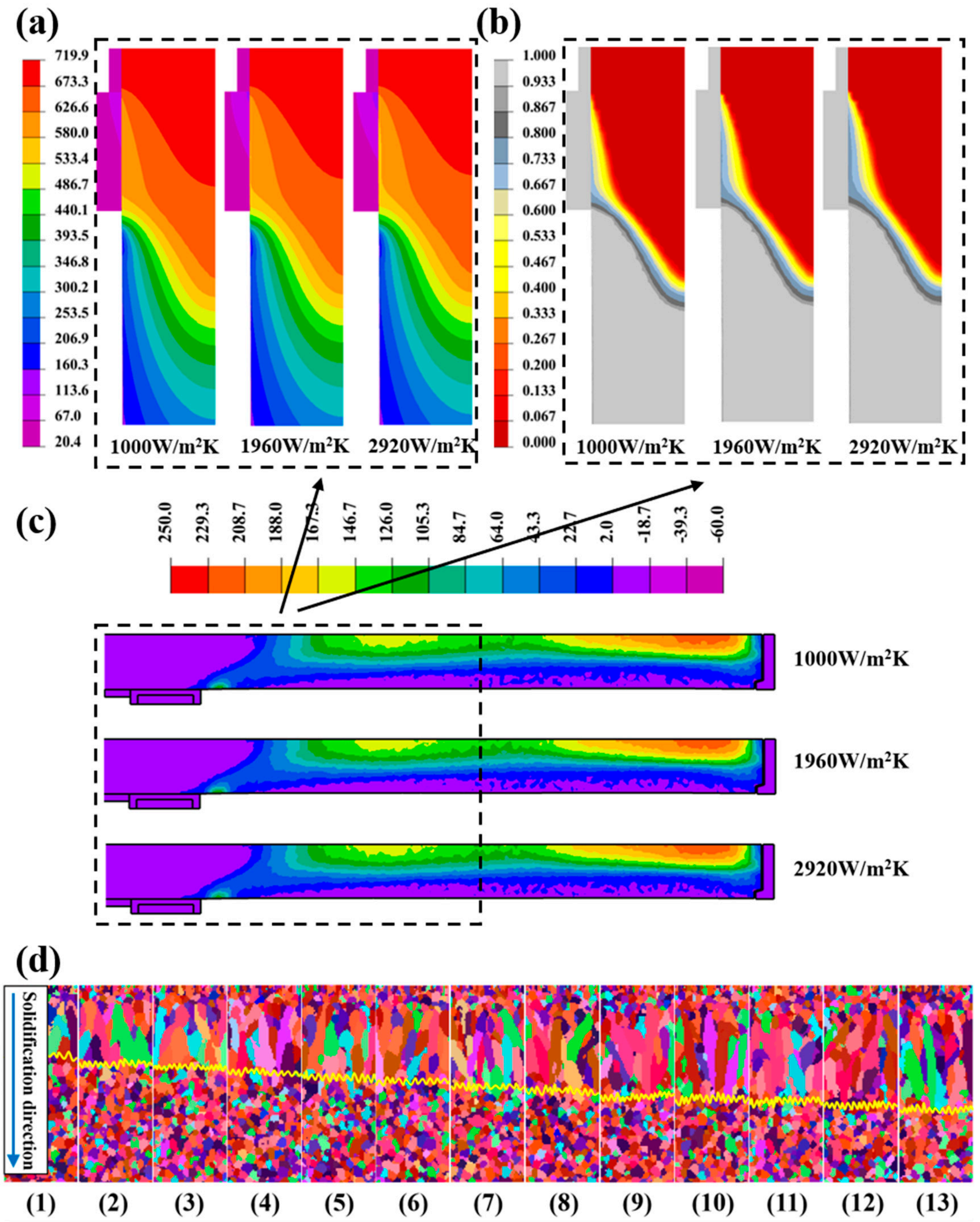

Figure S3. Contour maps of different PCIs on DC casting: (a) temperature field, (b) solid fraction, (c) stress field, (d) solidified structure.

Figure S4 presents the contour maps results regarding the impact of different secondary cooling water flow rates on the temperature field, stress field, and microstructure of the DC cast billet.

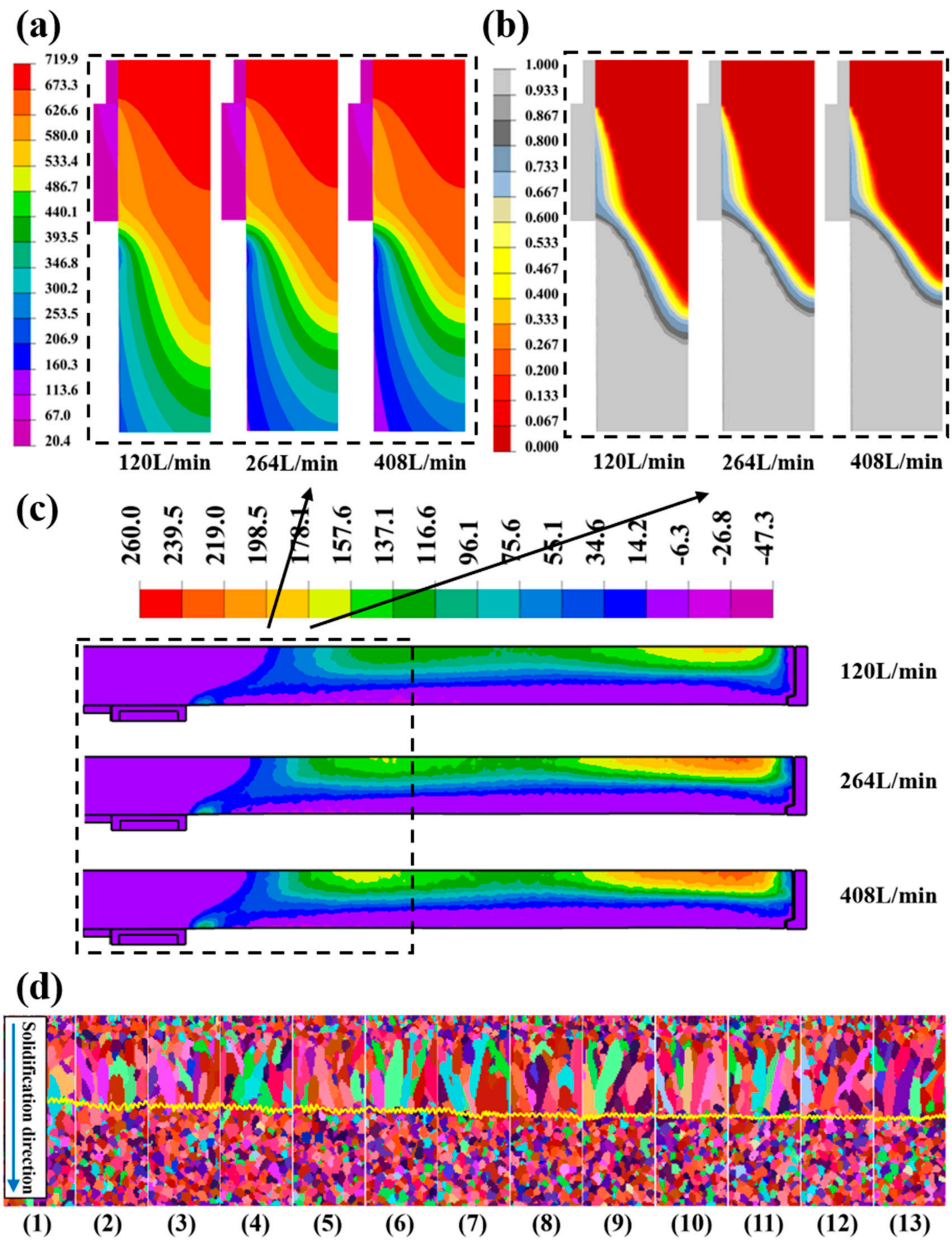

Figure S4. Contour maps of different SCWFRs on DC casting: (a) temperature field, (b) solid fraction, (c) stress field, (d) solidified structure.

Figure S5 presents the quantitative results of different process parameters on hot cracking susceptibility for DC casting.

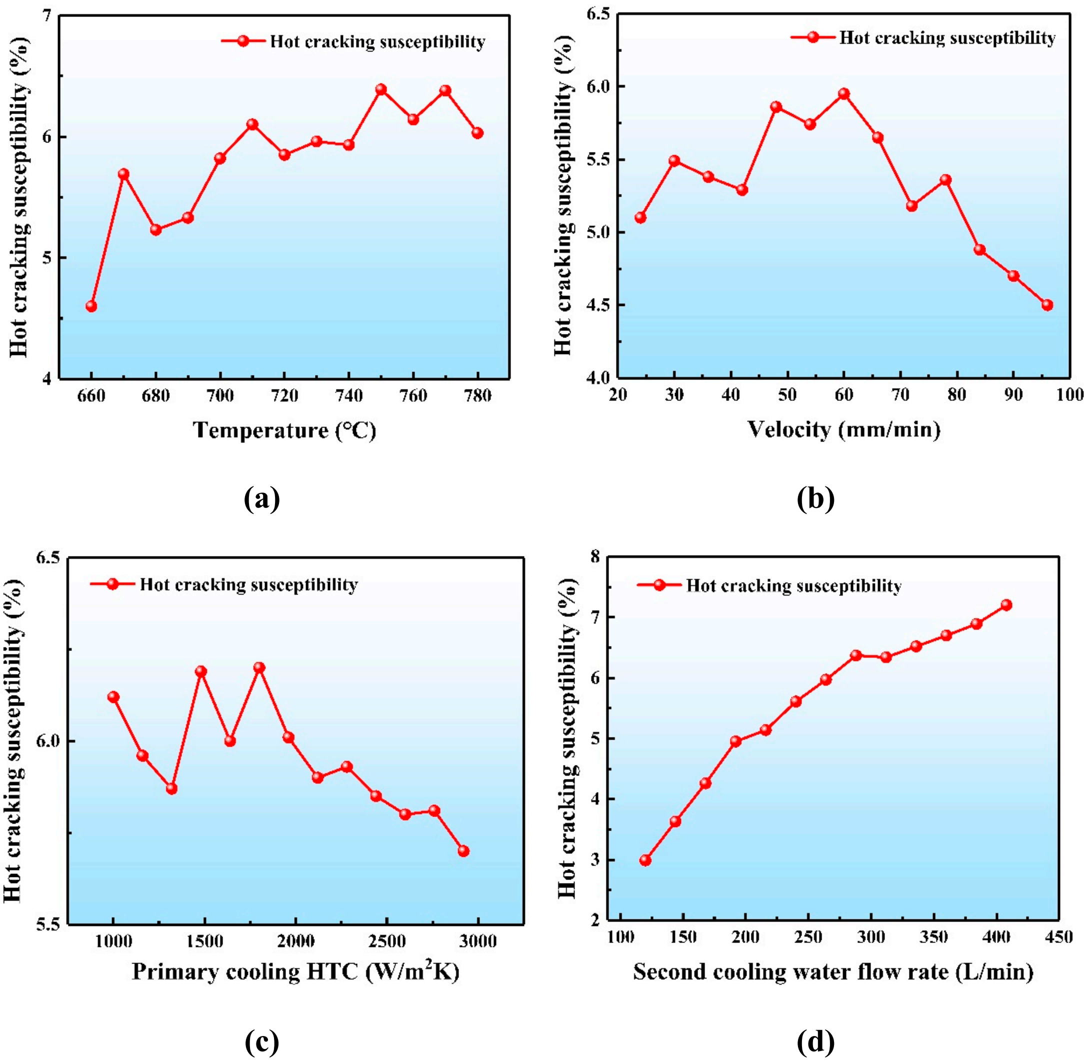

Figure S5. Quantitative results of different process parameters on HCS for DC casting: (a) different pouring temperatures, (b) different casting speeds, (c) different primary cooling intensities, (d) different second cooling water flow rates.

Figure S6 presents the schematic of the location for measuring first principal stress. Figure S7 shows the schematic of the location for measuring microstructure.

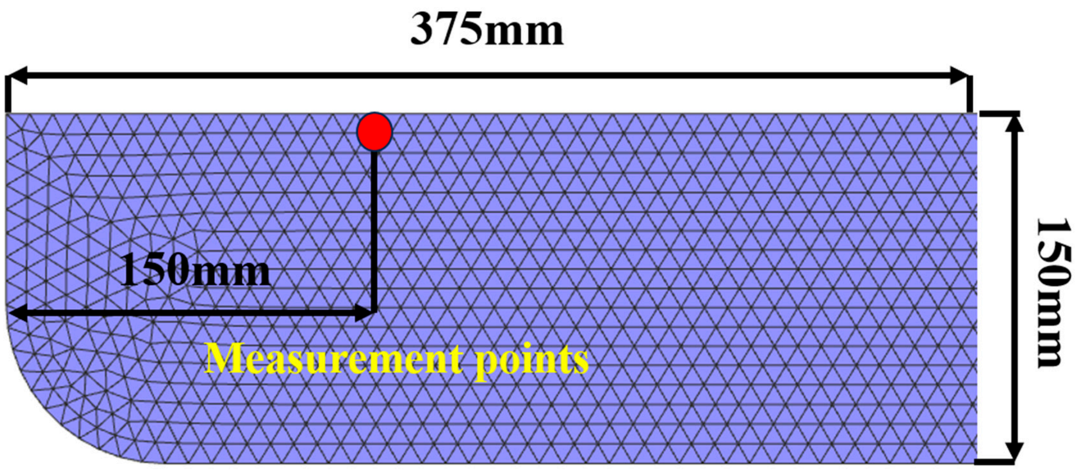

Figure S6. Schematic of the location for measuring first principal stress.

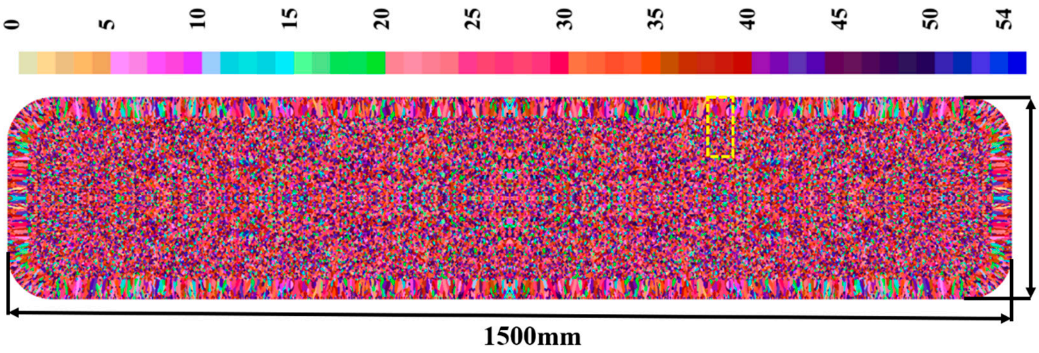

Figure S7. Schematic of the locations for measuring microstructure.
